# Species specific gene expression dynamics during harmful algal blooms

**DOI:** 10.1038/s41598-020-63326-8

**Published:** 2020-04-10

**Authors:** Gabriel Metegnier, Sauvann Paulino, Pierre Ramond, Raffaele Siano, Marc Sourisseau, Christophe Destombe, Mickael Le Gac

**Affiliations:** 1 French Research Institute for Exploitation of the Sea, Ifremer DYNECO PELAGOS, 29280 Plouzané, France; 2 CNRS, Sorbonne Université, UC, UaCh, UMI 3614, Evolutionary Biology and Ecology of Algae, Station Biologique de Roscoff, CS 90074, 29688 Roscoff, France; 3 CNRS, Sorbonne Université, UMR 7144, Station Biologique de Roscoff, CS90074, 29688 Roscoff Cedex, France

**Keywords:** Microbial ecology, Marine biology, Transcriptomics

## Abstract

Harmful algal blooms are caused by specific members of microbial communities. Understanding the dynamics of these events requires comparing the strategies developed by the problematic species to cope with environmental fluctuations to the ones developed by the other members of the community. During three consecutive years, the meta-transcriptome of micro-eukaryote communities was sequenced during blooms of the toxic dinoflagellate *Alexandrium minutum*. The dataset was analyzed to investigate species specific gene expression dynamics. Major shifts in gene expression were explained by the succession of different species within the community. Although expression patterns were strongly correlated with fluctuation of the abiotic environment, and more specifically with nutrient concentration, transcripts specifically involved in nutrient uptake and metabolism did not display extensive changes in gene expression. Compared to the other members of the community, *A. minutum* displayed a very specific expression pattern, with lower expression of photosynthesis transcripts and central metabolism genes (TCA cycle, glucose metabolism, glycolysis…) and contrasting expression pattern of ion transporters across environmental conditions. These results suggest the importance of mixotrophy, cell motility and cell-to-cell interactions during *A. minutum* blooms.

## Introduction

Micro-eukaryote communities are key components of the marine ecosystem. They enable carbon fixation, oxygen production and constitute the basis of marine food webs. In nutrient rich coastal ecosystems, transient but massive development of micro-eukaryotes leads to algal blooms with adverse consequences on both natural ecosystems and human activities. Examples of negative impacts include the development of anoxic conditions in sheltered bays, the production of compounds damaging fish gills, and of toxins bio-accumulating along the food webs and potentially lethal to humans^1,2^. The reporting of these harmful algal blooms (HAB) has dramatically increased over the last decades, although it remains unclear if this trend is linked to human mediated global environmental changes, or reflects the improvement of HAB monitoring across the globe^2^. During HAB events, only a single or a few specific species, co-occurring with non-problematic species within communities, are responsible for the negative impacts. Understanding the dynamics of these harmful events requires comparing the strategies developed by the problematic species to cope with environmental fluctuations to the ones developed by the other members of the community. It may be addressed by monitoring species successions in natural communities, as for example performed using metabarcoding analyses of natural communities^3,4^, but such approaches only focus on the structural composition of the communities (who is present in the community?). By simultaneously quantifying the expression of thousands of genes, transcriptomic approaches enable to monitor the cellular strategies of various organisms. When applied to natural communities, such approaches offer the promise to decipher the cellular strategies developed by the various species in response to the same environmental conditions (what are the community members doing?). Thanks to the next generation sequencing revolution, it is now easy to generate community wide transcriptomic datasets, and several such studies have already been published, starting with prokaryotic communities^5–14^. Similar approaches focusing on marine micro-eukaryote communities have been developed more recently. One of the main obstacles had been the lack of reference genomes or transcriptomes for the vast majority of marine unicellular eukaryotes^15^. Thanks to the Marine Microbial Eukaryotic Transcriptome Sequencing Project (MMETSP^16^), that allowed the generation of reference transcriptomes for more than 200 species, a major step has been taken. Following this initial effort, several meta-transcriptomic studies specifically focusing on unicellular eukaryotes have been developped^17–27^. In these studies, meta-transcriptomic profiling has been used to monitor the response of natural micro-eukaryote communities following the addition of iron^17,24^, of nutrient rich deep sea water^18^ to surface communities, or the incubation of deep-water communities in sunlit conditions^23^. Meta-transcriptomic approaches have also been developed to investigate the variability of global gene expression at different depth^21^, at local^20^ or global^22^ geographical scales, as well as through time^19^. Most of these studies focused on the contrasting patterns of expression among major micro-eukaryote phyla such as diatoms, dinoflagellates, haptophytes, and ciliates^17,18,20–23^ with some exceptions that explicitly focused on differential expression patterns between co-occurring Diatoms^19,24^. Some studies specifically focused on species involved in HAB. It was the case during a bloom of *Heterosigma akashiwo* during which genes potentially involved in phosphorous uptake, carbon fixation but also mixotrophy displayed variable expression^25^. In a different system, experimental addition of nutrients triggered transcriptional responses, especially regarding genes known to be associated with phosphorus deficiency, in natural populations of the brown tide causing species *Aureococcus anophagefferens*^27^. Most of the studies presented above were specifically interested in the transcriptomic response of the communities to specific environmental variation such as iron^17,24^ or nutrient^18,19,23,27^ limitations and manipulated the natural communities to focus on the effect of these environmental fluctuations. Here, our goal was rather to identify without *a priori* the cellular functions displaying high gene expression variability in the field and to correlate them with environmental fluctuations. This approach was developed as a mean to raise new hypotheses regarding how different species belonging to the same community cope with environmental fluctuations.

In the present study, we used a meta-transcriptomic approach to investigate the expression dynamics of twelve species co-occurring during blooms of the harmful dinoflagellate *A. minutum*. The expression dynamics was investigated at the species level to compare the “metabolic” status of the harmful species to the one of the co-occurring species. Targeting the species level is essential to identify the specificities of the problematic species and gain new insights on the factors that may enable such species to transiently dominate micro-eukaryote communities. *Alexandrium minutum* is involved in paralytic shellfish poisoning (PSP) blooms worldwide^28^. Bloom initiation is assumed to begin with the excystment of resting cells that may be regulated by temperature, light and oxygen concentration^28,29^. Bloom development seems to be strongly constrained by hydrodynamics (characterized by both tide, wind and river flow^28,30–33^). Nutrient concentration has also been identified as a key factor controlling bloom dynamics^31,34,35^. The factors responsible for the ending of the bloom are more difficult to define. Among possible processes involved, several studies have highlighted significant roles of nutrient limitation^30,36,37^, competition^33^, predation^38,39^, and parasitism^40^.

The blooms were sampled during three years (2013–2015) in the Bay of Brest (France). The generated dataset was analyzed to investigate whether the various species within the community display similar expression dynamics in response to environmental fluctuations or whether each species display its own expression dynamics. More specifically, we asked the following questions: 1. Can we correlate expression dynamics to environmental fluctuations? 2. Do the different species display similar expression dynamics in response to environmental fluctuations? 3. Does *A. minutum* display specific expression dynamics?

## Results

### Determining the most abundant species based on gene expression

**Figure 1:**
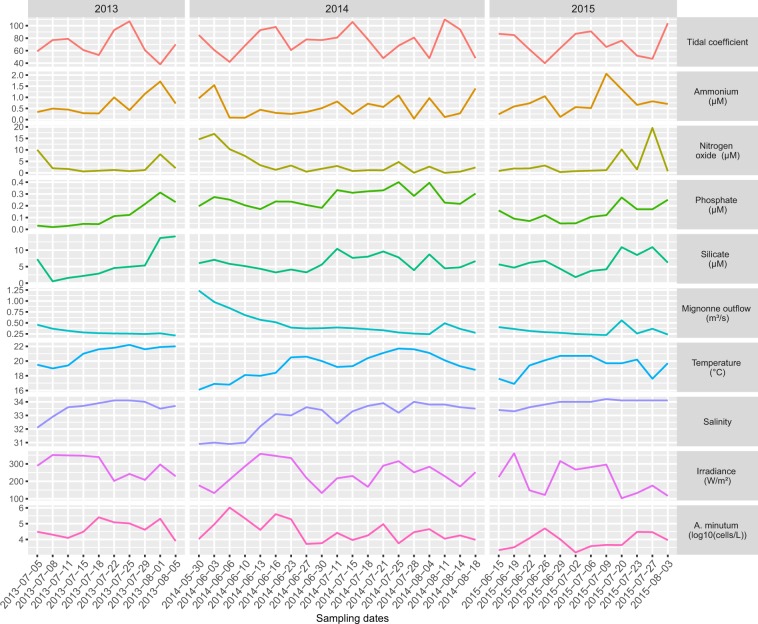
Environmental parameters. For each sampling date, values of nine abiotic environmental parameters and *A. minutum* cell densities.

**Figure 2:**
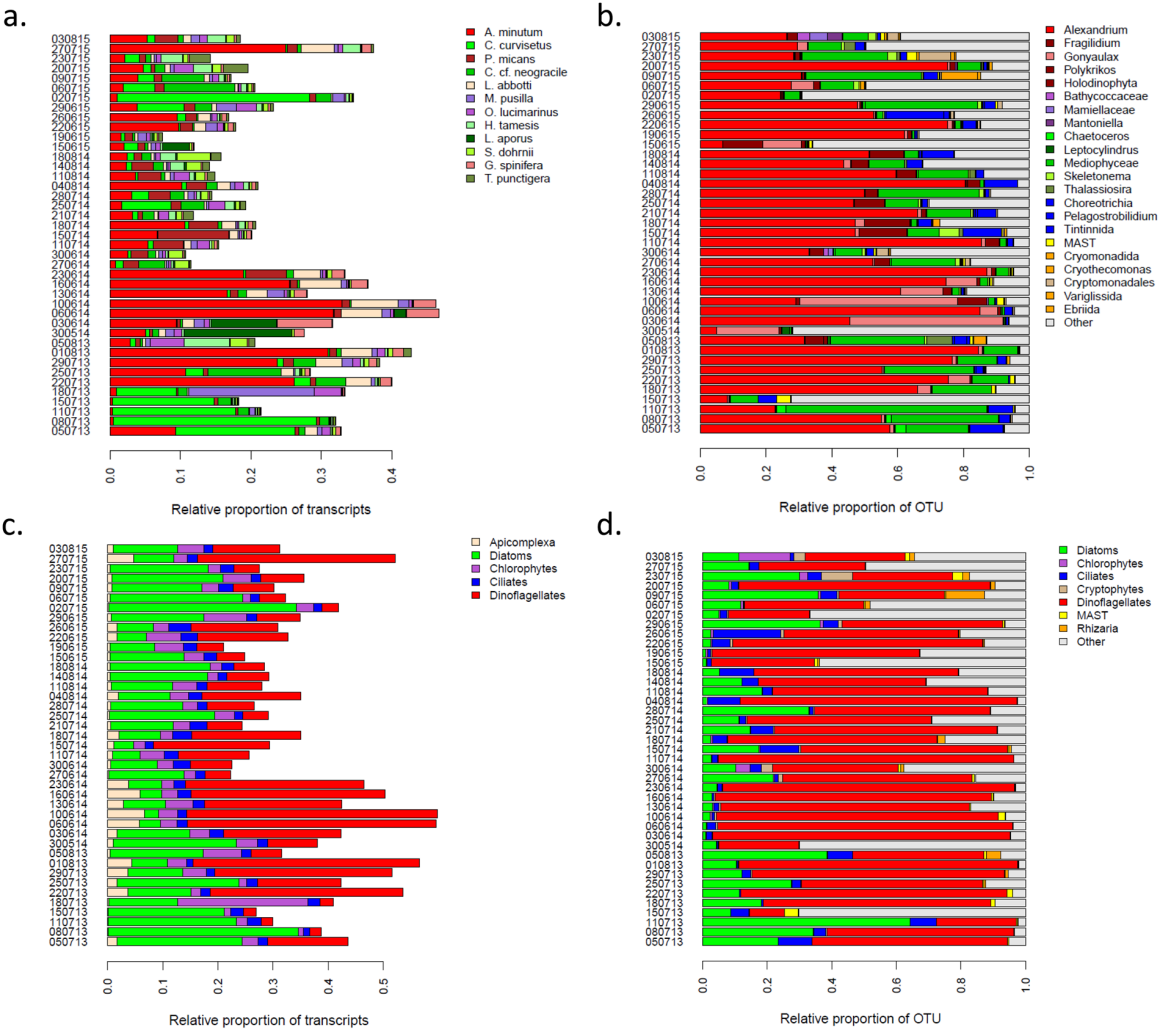
Relative abundances of the members of the sampled communities for each sampled time point. Panels a. and c. indicate the relative abundance of transcripts (RAT, see methods) for each a. species, and b. phylum. Panels b. and d. indicate the relative abundance of OTU based on metabarcoding data for each b. genera, and c. phylum.

During three consecutive years, an *in situ* survey (Fig. 1; Supplementary Table S1) of *A. minutum* blooms was performed in the Bay of Brest (France), resulting in 41 meta-transcriptomic datasets (a total of 16.10^8^ reads). The environmental reads were aligned to a meta-reference (MetaRef2; see methods) composed of the reference transcriptomes of 219 different species. A total of 12 species displayed a maximum Relative Abundance of Transcripts (RAT; see methods) higher than 0.03 and were specifically analyzed. Out of these 12 species, there were six large diatoms (*Chaetoceros curvisetus*, *C. cf. neogracile*, *Helicotheca tamesis*, *Leptocylindrus aporus*, *Skeletonema dorhnii*, and *Thalassiosira punctigera*), three dinoflagellates (*A. minutum*, *Gonyaulax spinifera*, *Prorocentrum micans*), two small (<2 µm) chlorophytes (*Micromonas pusilla*, and *Ostreococcus lucimarinus*) and one apicomplexan parasite of free-swimming tunicates (*Lankesteria abbotti*). For these 12 species, the total number of reads ranged from 17.10^5^ to 29.10^7^, representing between 1‰ and 17.4% of the analyzed reads for *P. micans* and *A. minutum*, respectively (Supplementary Table S2). The reads aligning to the 12 species added up to a total of 41.10^7^ reads, representing 25% of the environmental reads. The RAT of the 12 species was highly dynamics across time points (Fig. 2a). Without surprise, the transcripts of *A. minutum*, were especially abundant with 31 sampling times displaying a RAT > 0.02 and a maximum RAT = 0.33. Although RAT maybe taken as an estimation of the relative abundance of a species in the community, it was related to the absolute *A. minutum* cell densities in the samples (Spearman’s Rho= 0.60; Supplementary Fig. 1a). Three species often displayed a RAT > 0.02 and often reached RAT > 0.1. This was the case for *C. curvisetus* (14 samples with a RAT > 0.02, max RAT = 0.29) and *C*. cf. *neogracile* (9 samples with a RAT > 0.02, max RAT = 0.1), as well as *P. micans* (11 samples with a RAT > 0.02, max RAT = 0.1). Two species, *L. aporus* (3 samples with a RAT > 0.02, max RAT = 0.15) and *M. pusilla* (3 samples with a RAT > 0.02, max RAT = 0.18) very punctually displayed high RAT. Three others, *H. tamesis* (7 samples with a RAT > 0.02, max RAT = 0.07), *O. lucimarinus* (6 samples with a RAT > 0.02, max RAT = 0.05), and *L. abbotti* (10 samples with a RAT > 0.02, max RAT = 0.07) often displayed RAT > 0.02, but never reached high RAT. Finally, the last three species considered, S. *dorhnii* (3 samples with a RAT > 0.02, max RAT = 0.05), *T. punctigera* (2 samples with a RAT > 0.02, max RAT = 0.04), and *G. spinifera* (4 samples with a RAT > 0.02, max RAT = 0.08) displayed a RAT > 0.02 in a few samples and never reached high RAT.

Performing the RAT analysis at the scale of the major phylum clearly indicated that the aligned reads mainly belonged to diatoms and dinoflagellates in variable proportions and to a lesser extent to ciliates, chlorophytes, and Apicomplexa (Fig. 2c). The 12 species explained a very large proportion of the RAT observed at the phylum level, suggesting that the expression dynamics at the phylum level was mainly due to a few abundant species rather than to a multitude of rare species.

### Comparison with metabarcoding

In parallel to the meta-transcriptomic approach, the composition of the micro-eukaryote community was characterized using a metabarcoding approach (Fig. 2b and d). When comparing the two datasets, the same genus were often identified with the two approaches. This was indeed the case within dinoflagellates for the genus *Alexandrium*, but also *Gonyaulax*, and within the diatoms for the genus *Chaetoceros*, *Leptocylindrus*, *Skeletonema*, *Thalassiosira*. Some of the results that might, at first sight, appear as not entirely compatible among the two datasets probably simply reflected a difference in taxonomic resolution of the meta-transcriptomic and meta-barcoding approaches. For instance, *P. micans* and *Chaetoceros* in the meta-transcriptomic dataset might correspond to the Holodinophyta and Mediophyceae OTUs in the meta-barcoding dataset, respectively. A major difference between the two datasets was the amount of relative abundance of transcripts and of OTU captured by the two datasets. While the 12 species corresponded on average to a RAT of 0.24 across samples (considering the raw data instead of the normalized proportion, the average proportion of aligned reads was 0.43), the average relative proportion of OTU that could be attributed to micro-eukaryote was 0.78 (the category Other corresponding to a mix of metazoan, land plant, unresolved eukaryotes and unassigned reads). This difference was mainly explained by the same species representing a very different relative abundance of transcripts and OTU. This was extremely clear for *Alexandrium*, representing an average RAT of 0.09 (average proportion of aligned reads of 0.17) while displaying an average relative proportion of OTU of 0.50. There was a better correlation between RAT and *A. minutum* cellular densities (Spearman’s Rho = 0.60; Supplementary Fig. 1a), than between *A. minutum* RAT and *A. minutum* relative OTU abundances (Spearman’s Rho = 0.48; Supplementary Fig. 1b), or between *A. minutum* relative OTU abundances and *A. minutum* cellular densities (Spearman’s Rho = 0.50; Supplementary Fig. 1c).

Another important difference was highlighted when considering the phylum level. While in term of expression, the average of the ratio between RAT of dinoflagellates and diatoms was 1.1 (2.4 considering raw reads), when considering the metabarcoding data, the average of the ratio rose to 4.2, indicating that in the same community rRNA/mRNA ratio might be four times higher for Dinoflagellates compared to Diatoms.

### Gene expression dynamics *in situ*

**Figure 3:**
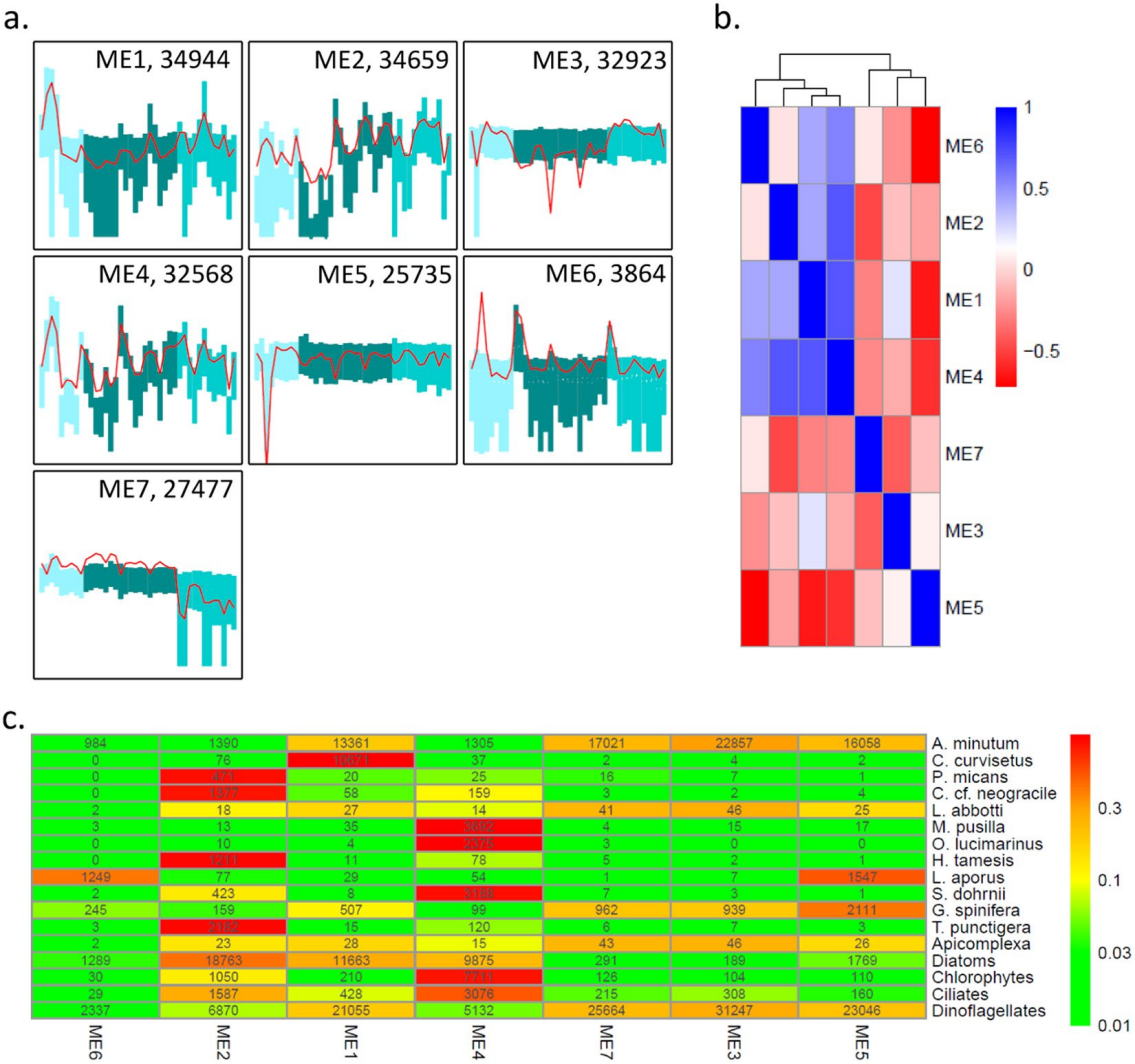
Modules of transcripts displaying similar expression dynamics across samples. a. the 7 identified expression modules. The 40 samples are on the x-axis, the shade of the bars represent the three years (2013, 2014, 2015). The y-axis indicates the level of expression (vst transformed raw-counts, range (−7,12)), the red lines indicate the expression dynamics of the eigengenes, the bars range from the first to the third quartile of the expression levels. The number of transcripts belonging to each module is indicated, b. Heatmap representing the Pearson correlation coefficient among the 7 expression modules. c. Heatmap indicating how the transcripts of 12 species as well as of the main phylum are distributed in the 7 expression modules. The color scale is based on the proportion of transcripts from each species/phylum assigned to each module. Numbers indicate the number of transcripts in each module.

A total of seven modules regrouping between 3,864 and 34,944 transcripts displaying similar expression dynamics across samples were identified (Fig. 3a). The expression dynamics of these modules across samples ranged from relatively stable (Modules ME3, ME5, ME7) to highly dynamics (ME2, ME4). The eigengenes of four modules had a slightly correlated expression dynamics (ME1, ME2, ME4, ME6) while the three others appeared completely independent (ME3, ME5, ME7) (Fig. 3b). More specifically, the transcripts belonging to ME1 tend to display high expression at the beginning of the 2013 bloom and then around the middle of the 2015 bloom. The transcripts belonging to ME2 were less expressed during 2013 and at the beginning of the 2014 bloom and more expressed at the end of the 2014 bloom and during 2015. The ME3 transcripts were slightly more expressed during 2013 and 2015 than during 2014. The ME4 transcripts displayed fluctuating expression during each of the three years. The ME5 transcripts displayed a very low level of expression at a single sampling date (July 11^th^ 2013). ME6 transcripts were more expressed at the beginning of 2014 and 2015. Finally, ME7 transcripts were less expressed during 2015.

The distribution of the transcripts of the 12 species in the seven modules was highly heterogeneous, and was of course strongly linked to the RAT dynamics described above (Fig. 3c). Five of the modules were to great extent composed of transcripts belonging to the 12 species (>64% of the transcripts of ME1, ME3, ME5, ME6, ME7); while in the two remaining modules their proportion was considerably lower (21% and 34% of ME2, and ME4). Eight of the species of interest had more than 85% of their transcripts in a single module. This was the case of *C. curvisetus* (99% of the transcripts in ME1), *P. micans* (87% of the transcripts in ME2), *C. cf neogracile* (86% of the transcripts in ME2), *M. pusilla* (98% of the transcripts in ME4), *O. lucimarinus* (99% of the transcripts in ME4), *H. tamesis* (93% of the transcripts in ME2), *S. dohrnii* (88% of the transcripts in ME4), and *T. punctigera* (93% of the transcripts in ME2). For three species, a non-negligible proportion of transcripts were found in more than one module. This was the case of *A. minutum* displaying more than 900 transcripts in each of the seven modules and more than 20% of its transcripts in each of four modules (ME1, ME3, ME5, ME7). Transcripts from ME1, ME2 and ME4 were less expressed when *A. minutum* cells were abundant, while there was a tendency of transcripts from ME7 to be more expressed when *A. minutum* cells were abundant. The level of expression of transcripts from ME3, ME5 and ME6 was not related to *A. minutum* cell densities (Supplementary Fig. 2).

Two other species displayed numerous transcripts in several modules: *L. aporus* displayed 42% and 52% of its transcripts in ME5, and ME6 and *G. spinifera* had more than 18% of its transcripts in each of four modules (ME1, ME3, ME5, ME7). The last species, *L. abbotti* displayed very few transcripts in all the modules and was not further analyzed.

Focusing on the transcripts at the phylum level (Fig. 3c), we note that three modules were mainly composed of transcripts from dinoflagellates and diatoms (ME1, ME2, ME6) in mixed proportions. One module was composed of transcripts from diatoms and dinoflagellates but also from chlorophytes and ciliates (ME4). The last three modules were mostly composed of transcripts from dinoflagellates (ME5, ME3, ME7;>89% of the transcripts). As stated above, two modules (ME2, ME4) were composed of transcripts not belonging to the 12 species specifically analyzed. In ME2, more than 40% of the transcripts aligned to other diatoms reference transcriptomes. In ME4, the transcripts mostly corresponded to other diatoms (20%), dinoflagellates (11%), but also ciliates (10%).

### Relationships between gene expression dynamics and environmental factors

**Figure 4:**
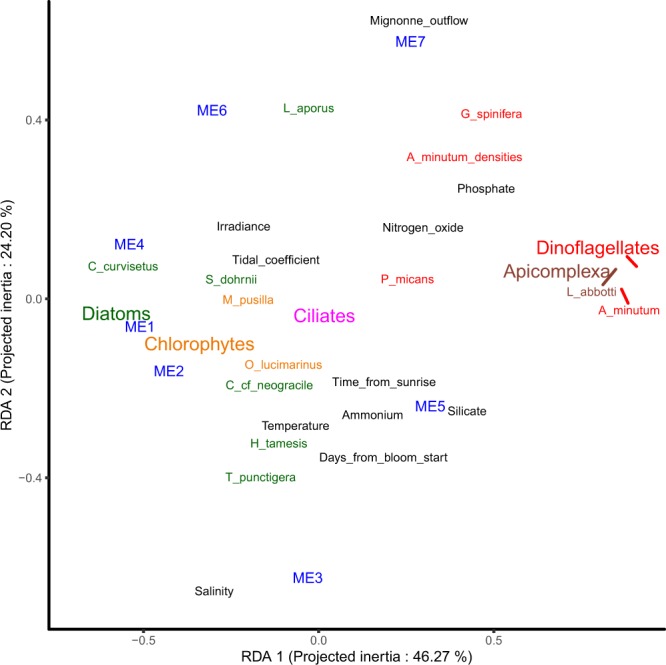
Representation of the two first axis of the RDA led between the gene expression modules and environmental factors. The eigengene of the expression modules (ME1 to 7) are plotted with the contribution of 11 explicative abiotic environmental variables (in black), of *A. minutum* cell densities and of the relative abundance of transcripts (RAT) of diatoms (green), dinoflagellates (red), chlorophytes (orange), ciliates (pink), and Apicomplexa (brown).

A set of abiotic factors was used to characterize the abiotic environment encountered by the sampled micro-eukaryote communities (Fig. 1). The expression dynamics of the seven modules were correlated with these abiotic factors as well as with absolute *A. minutum* cell densities and with the RAT (taken as an indicator of the relative abundance of the species/phylum within the community). Focusing on *A. minutum*, absolute cell densities were higher when nitrogen oxide (nitrate, nitrite) and phosphorous displayed higher concentrations (Fig. 4). As already noted above, *A. minutum* transcripts were especially abundant in four modules. Interestingly, ME1 regrouped transcripts more expressed when absolute and relative *A. minutum* abundances tend to be low in nutrient poor environmental conditions and when Diatoms tend to be relatively abundant. Transcripts in ME3 and ME7 tend to display high expression in contrasting environmental conditions with high salinity and high river flow, respectively. Finally, ME5 transcripts tend to be more expressed when Diatoms were relatively rare.

More generally, and as already suggested by the analyses presented above, a large portion of the expression variance was linked to fluctuation of relative abundance of the species composing the community (Fig. 4). The first axis strongly separated the expression modules highly expressed in a nutrient rich (phosphate, nitrogen oxide, silicate) environment with a high relative abundance of dinoflagellates (ME5, ME7, Fig. 4) and in a nutrient poor environment with high relative abundance of diatoms (ME1, ME2, ME4, Fig. 4). The second axis mainly separated modules displaying dynamic expression levels in relation with river outflow and associated salinity fluctuations.

### Functions of the transcripts displaying dynamic expression

**Figure 5:**
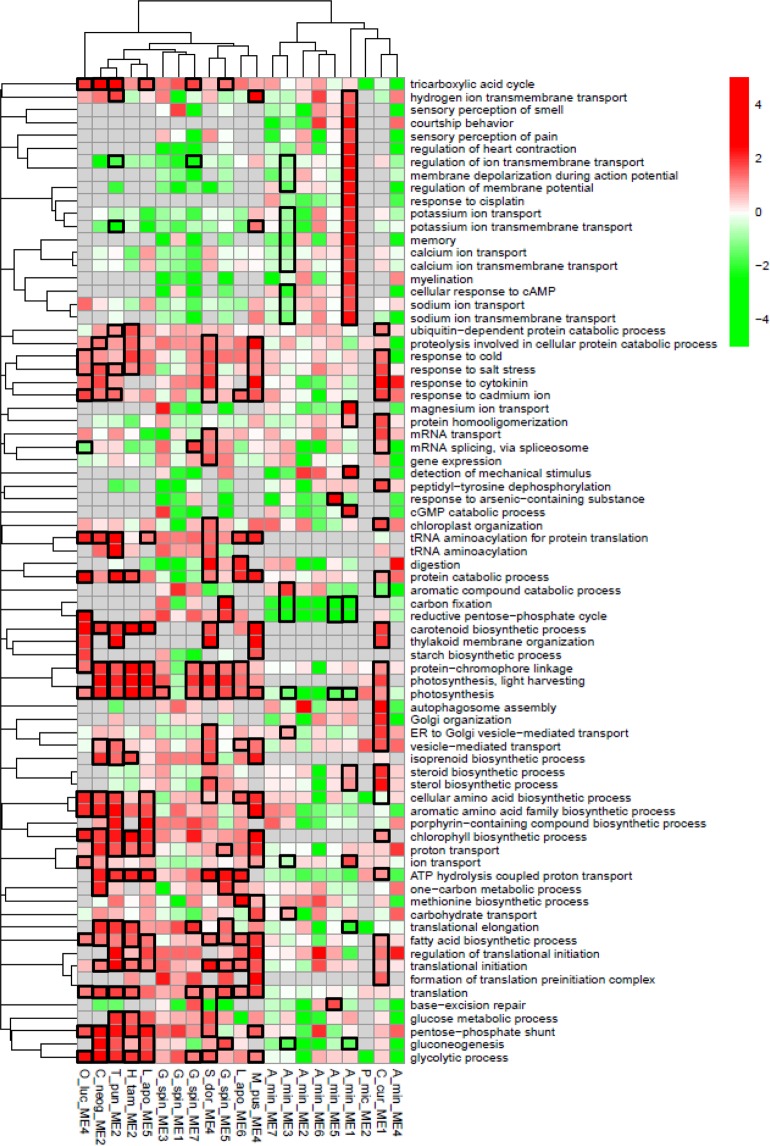
Over-representation of Biological Process GO terms in each species in each expression module. Heatmap colors indicate the odd-ratio for each GO term (GO displaying less than 20 transcripts were not analyzed and are in grey). GO terms were clustered based on overlap between GO (see Material and Methods for details). Black squares indicate q-values < 0.1.

The functions of the transcripts grouped into expression modules were investigated at the species level (see Material and Methods for details; Fig. 5). A total of 76 Gene Ontology (GO) biological processes were identified as displaying a species specific transcript enrichment in at least one expression module.

1. *Alexandrium minutum* displays strong species specific functional enrichment
  Overall, compared to the other species considered, *A. minutum* displayed a strong depletion of transcripts related to photosynthesis, carbon fixation, reductive pentose-phosphate cycle and gluconeogenesis. This contrasting pattern was also visible in specific expression modules. For instance, in ME3, photosynthesis related transcripts were over-represented in *G. spinifera* but under-represented in *A. minutum*. Similarly, in ME1, photosynthesis related transcripts were over-represented in *C. curvisetus* but under-represented in *A. minutum*.
  *Alexandrium minutum* also displayed a contrasting over-representation pattern across expression modules. In ME1, corresponding to transcripts more expressed when the community was dominated by Diatoms and *A. minutum* was less abundant, numerous ion transport related functions were over-represented. These same functions were strongly under-represented in ME3, corresponding to transcripts more expressed when *A. minutum* was more abundant in an environment characterized by high river flow.
2. For the other members of the community, the transcripts belonging to different expression modules were related to different biological processes.
  Two other species displayed numerous transcripts in different expression modules. This was the case of *L. aporus* in ME5 and ME6. Of special interest was the over-representation of transcripts involved in photosynthesis, TCA cycle, as well as glycolysis/pentose phosphate shunt in ME5, while transcripts involved in digestion/protein catabolism were over-represented in ME6 (transcripts involved in photosynthesis were also over-represented in ME6, but to a lesser extent). The Dinoflagellate *G. spinifera* displayed slight differences among over-represented functions across four expression modules. We may for instance note the over-representation of photosynthesis related functions in ME3, 5, and 7, but not in ME1.
3. Within a given expression module, the transcripts belonging to different species sometimes corresponded to the same biological processes
  In ME1, *A. minutum* and *C. curvicetus* displayed over-representation of sterol/steroid biosynthesis transcripts. In ME2, three Diatoms (*H. tamesis*, *T. punctigera*, *C. cf. neogracile*) displayed extremely similar patterns, with high expression of photosynthesis, but also glycolysis, and fatty acid biosynthesis. In ME4, two chlorophytes (*O. lucimarinus, and M. pusilla*) and a Diatom (*S. dohrnii*) species displayed a partially similar pattern, with over-representation of photosynthesis, glycolysis, and pentose-phosphate related transcripts in the three species.
4. Species belonging to the same phylum did not necessarily display similar biological process over-representation patterns

The over-represented biological processes were compared among species belonging to the same phylum. The diatom species tend to display a constant over-representation of transcripts related to photosynthesis but also to a lesser extent to fatty acid biosynthesis. Other central biological processes, such as the ones related to the TCA cycle, glucose metabolism, glycolysis, gluconeogenesis, and pentose phosphate shunt displayed a contrasting pattern across species. The species *C. curvisetus* displayed an especially contrasting pattern, with over-representation of transcripts related to sterol/steroid metabolism, to autophagosome assembly and Golgi organization. The two chlorophytes displayed a similar pattern with over-representation of photosynthesis, glycolysis, and pentose-phosphate and starch biosynthesis related transcripts. Finally, as already suggested above, *A. minutum* displayed a contrasting over-representation pattern compared to the other two dinoflagellates.

## Discussion

During a three year *in situ* survey of the toxic dinoflagellate *A. minutum*, meta-transcriptomic datasets were used to investigate the species specific gene expression dynamics of the different species dominating this micro-eukaryote community. A major challenge to infer species specific gene expression dynamics is to ensure the specificity of the alignment of the environmental reads on the reference transcriptomes. Strong homology between orthologous genes may interfere with the robustness of the alignment. To cope with such issue, we propose to align environmental reads to a meta-reference composed of a maximum of species specific transcriptomes, only keeping one species per genus when several species belonging to the same genus display extremely similar relative abundance dynamics, and discarding environmental reads aligning to several reference transcripts. Thanks to the availability of the MMETSP dataset^16^ we used a meta-reference composed of around 200 species reference transcriptomes. Although the development of this dataset is a major breakthrough, we may wonder at what point it is sufficient to follow species specific expression dynamics of entire micro-eukaryote communities. As a matter of comparison, meta-barcoding approaches aiming at characterizing the composition of communities use reference databases corresponding to several thousands of OTU^41^. Reassuringly, after comparing meta-transcriptomic and meta-barcoding approaches, results were congruent regarding the species identified as dominating the sampled communities. However, there were striking differences regarding the proportion of the environmental sequences aligning to the references. While the proportion of meta-barcoding reads that could be assigned to the abundant OTU was often higher than 0.8, the proportion of meta-transcriptomic reads assigned to the abundant species was often lower than 0.3. Making our alignment parameters less strict (and especially allowing for a single read to align to several transcripts), or performing phylum wide analyses had little influence (a few percent) on this proportion. In other analyses of meta-transcriptomic datasets, the proportion of reads taxonomically assigned was around 0.5^18–20,22^. Several kinds of explanations maybe possible, the first one involves the quality of some reference transcriptomes. For example, *P. micans* reference transcriptome is extremely short and incomplete, precluding global expression profiling for this species. Second, intraspecific diversity may also play a role. Reference transcriptomes often result from the sequencing of a single strain for a given species, precluding gene expression analysis at the pan-genome (the total set of genes found in a given species) scale. For example, about 60% of the baker yeast (*Saccharomyces cerevisiae*) genes belong to the core genome^42^ (the gene set shared by all strains of a given species). For the haptophyte *Emiliania huxleyi*, the core genome corresponds to ~80% of the genome^43^. For the other marine micro-eukaryotes this proportion is virtually unknown. Third, the number of cryptic species within micro-eukaryotes is probably extremely high. As an example, following the discovery of the toxicity in the Diatom genus *Pseudo-nitzschia*, the number of species rose from about 15 in the 80s^44^ to 51 nowadays^45^. If closely related but nevertheless distinct species are present in the sampled community and in the meta-reference, the expression profiling would be extremely partial, as it would only be possible for the orthologous genes (the genes represented in the genomes of the two closely related species). For example, in *Pseudo-nitzschia*, the number of orthologous transcripts identified in three species represented between 20% and 40% of each species reference transcriptome^46^. Finally, a last but potentially extremely important factor that could affect the expression profiling in natural communities is the presence of transcripts expressed *in situ* but barely if at all in the artificial experimental environments that were used to obtain the reference transcriptomes. It would, of course, be impossible to characterize the expression dynamics of such transcripts, and solving this problem is far from being straightforward. Indeed, if solving the first three issues presented above is “only” a matter of improving the quality of the sequencing or sequencing more strains or more species; characterizing transcripts only expressed *in situ* for organisms displaying a genome too big to be sequenced is highly problematic. Some of our results tend to show that this may be a non-negligible problem. Considering *A. minutum*, for which the reference transcriptome was obtained using several strains isolated locally^47^, and often representing about 50% of the OTU, the number of reads that aligned to the reference transcriptome was at time very low (see for instance the second half of the season 2014, Fig. 1). It tends to indicate that this reference transcriptome only enabled a partial characterization of *A. minutum* expression.

As discussed above, performing species specific gene expression dynamics within natural communities is not without causing problems, and analyzing expression dynamics at the phylum level may seem like an interesting alternative^17,18,20,21^. However, such approaches make important implicit assumptions which may not hold for all communities. The first implicit assumption is that the various species belonging to the same phylum are more or less ecologically redundant^48^, that they respond more or less similarly to environmental fluctuations, and that as a result global expression patterns are much more similar within than between phylum. Here, and as already noted in previous studies^23,24^ we saw it is not necessarily the case. For instance, diatom expression profiles are sometimes more similar among species belonging to different phylum than among species of the same phylum. Within dinoflagellates, the difference between *G. spinifera* and *A. minutum* regarding the expression of photosynthesis related transcripts is especially remarkable. The second assumption is that the community is composed of a multitude of co-occurring species and that the community wide expression dynamics is mainly driven by changes in expression within the co-occurring species and not by major modifications in terms of community composition. This second assumption does not hold in the community investigated in the present study as a great proportion of this community is composed of a few dominant species displaying major relative abundance fluctuations across samples. For instance, diatom transcripts are abundant in three modules of expression, but the species mainly contributing are different from one module to the other, meaning that shifts in expression patterns is driven by the succession of different species.

The expression profile of the harmful species *A. minutum* strongly contrasted with the expression profiles of the other species present in the community. The first major difference was related to transcripts linked to photosynthesis. Transcripts associated with this biological process tend to be over-represented in most species. It was the case in the present study but also in previous studies investigating diatom gene expression *in situ*^18,20^. On the contrary, *A. minutum* displayed a systematic under-representation of photosynthesis related transcripts, which might indicate that *A. minutum* did not heavily rely on photosynthesis *in situ*. Dinoflagellates^49,50^ and more specifically *Alexandrium* species^51^ develop mixotrophic strategies, and *in vitro* assays showed that mixotrophy may considerably improve *Alexandrium* growth rate^51^. Interestingly, the importance of mixotrophy was also suggested by gene expression patterns during blooms of the harmful species *Heterosigma akashiwo*^25^. The second major difference was related to ion (Na, Ca, K) transporters. In *A. minutum*, these transcripts displayed contrasting expression patterns across environmental conditions. More specifically, high expression levels were observed in environmental conditions characterized by a high relative abundance of diatoms in the community and low expression levels in environmental conditions characterized by high salinity that tend to occur more often toward the end of the blooming period. Ion transport may be related to at least two distinct types of ecologically relevant functions. First, it may be linked to cell motility^52^. For instance, ionic fluxes were shown to be involved in motile and sensory responses in *Chlamydomonas*^53–55^. In the genus *Noctiluca*, tentacle movements were shown to be initiated and regulated by calcium (and also Na^+^) transporters^56,57^. Second, ion fluxes may also be related to cell to cell interactions. While calcium signaling might not be extensively studied in dinoflagellates, a few reports in *Crypthecodinium cohnii* show that calcium fluxes modulation plays a role in cell cycle regulation in response to mechanical stimulation^58,59^. Channels regulating calcium concentration are known for transducing organized and controlled spatial and temporal sensory signals. In diatoms, calcium concentration elevations were shown to respond to mechanical stimuli^60^. In two divergent *A. minutum* groups of strains, genes involved in various calcium related functions were shown to be among the most divergent genetically within *A. minutum*^47^.

Finally, major shifts in expression patterns were identified when the environment was enriched in nutrients due to high river flow compared to conditions where river flow was lower and nutrients less abundant. However, the transcriptional response did not seem to be a direct consequence of nutrient fluctuation. Indeed, for *A. minutum* as well as for the other dominant species in the community, transcripts involved in nutrient uptake and metabolism were not identified as one of the major components of gene expression dynamics *in situ*. This contrasts with previous meta-transcriptomic studies^18,20,25,27^ that were able to correlate transcriptional shifts in genes known to be involved in nutrient uptake and metabolism with variation in nutrient concentration. One possible explanation for this difference may be that in the present system, nutrient concentration fluctuates moderately and rarely indicates nutrient depletion, while more extreme fluctuations were monitored in some previous studies^20,25^. In other studies, natural communities were subjected to major experimental amendment of phosphorous and/or nitrate^18,27^, that probably triggered the transcriptional responses.

## Conclusions

By analyzing *in situ* gene expression of the harmful dinoflagellate *A. minutum*, as well as of 11 co-occurring unicellular eukaryote species, during three consecutive summers, we showed that major gene expression changes involve the succession of different species in the community. The transcriptional shifts were strongly linked to the fluctuation of the abiotic environment and contrasted when river flow was high (nutrient rich, low salinity) and low (nutrient poor, high salinity). However, transcripts specifically involved in nutrient uptake and metabolism did not display extensive gene expression variation. Of special interest, the toxic species *A. minutum* displayed an extremely specific expression pattern. Compared to the other members of the community, this species showed a strong depletion of transcripts involved in carbon fixation and photosynthesis, questioning the trophic mode (autotrophy/mixotrophy) of *A. minutum* in the field. Still compared to the other members of the community, the expression of numerous ion transporters (Na, Ca, K) was highly variable in *A. minutum*. These transcripts may be related to ecological important functions such as cell motility or cell-to-cell interactions. However, major gene annotation effort has still to be made to take full advantage of meta-transcriptomic approaches and be able to connect *in situ* gene expression patterns to ecology relevant processes. In addition to the homology analyses, annotation will require coupling experimental and *in situ* based approaches to identify and validate molecular markers of specific ecologically relevant processes^61^.

## Material and Methods

### Sampling

During three consecutive summers (2013, 2014 and 2015), the micro-eukaryote community was sampled at a single site (48.350543, −4.292507) in the Bay of Brest (France). Water was sampled twice a week, when *A. minutum* concentration was superior to 10,000 cells.L^−1^. This threshold is considered as indicative of a potential toxicity risk by the French network for phytoplankton and phycotoxin monitoring (REPHY). Several environmental conditions were monitored for each sampling, including salinity, sea surface temperature, concentration of ammonia, nitrate, nitrite, phosphate and silicate (determined in the laboratory using a Seal Analytical AA3 HR automatic analyzer^62^), the number of days since the beginning of the bloom period (>10,000 cells.L^−1^), the time elapsed between sunrise and sampling, the tidal coefficient (http://maree.shom.fr/), the flow of the adjacent Mignonne river (http://hydro.eaufrance.fr/), and the irradiance at sea surface (http://www.meteofrance.com/) at the time of the sampling were also recorded (Fig. 1; Supplementary Table S1). Water samples (4.8 ± 1.3 (mean ± SD) liters; Supplementary Table S1) were filtered (20 µm) using a peristaltic pump, filters were frozen in liquid nitrogen and stored at −80 °C until RNA extraction. To prevent RNA degradation during thawing, RNA Later Ice (Fisher Scientific, Illkirch, France) was added before thawing for samples collected in 2013. In 2014 and 2015, samples were directly frozen with RNA Later (Fisher Scientific, Illkirch, France).

### Meta-transcriptomic

RNA was extracted following the Rneasy Plus Mini Kit (Qiagen, Courtaboeuf, France) recommendations, after ultra-sonication of the samples on ice (Vibra-cell 75115, Bioblock Scientific, Illkirch, France) for 10 seconds at 20% intensity. Libraries were prepared with the Truseq mRNA V2 kit (Illumina, Paris 3, France), and samples were sequenced at Get-PlaGe France Genomics sequencing platform (Toulouse, France) on Illumina HiSeq. 2000/2500 2*100 pb (2013 and 2014; Multiplex 10) and on HiSeq. 3000 (Illumina, Paris 3, France) 2*150 pb for 2015 samples (Multiplex 24). A total of 41 libraries were sequenced (10 in 2013, 19 in 2014 and 12 in 2015, see details in Supplementary Table S1).

Read quality was assessed using FastQC (http://www.bioinformatics.bbsrc.ac.uk/projects/fastqc/), and Trimmomatic^63^ (V. 0.33) was used to trim ambiguous, low quality reads and sequencing adapters with parameters ILLUMINACLIP: Adapt.fasta: 2 :30 :10 LEADING: 3 TRAILING: 3 MAXINFO: 135: 0,8 MINLEN: 80.

### Metabarcoding

Genomic DNA was extracted from 20 µm polycarbonate filters using DNA NucleoSpin Plant II extraction kit (Macherey‐Nagel, Hoerdt, France)^64^. The hyper‐variable V4 domain of the 18 S‐rDNA region was chosen as a barcode. Purified products were diluted to obtain equimolar concentrations before library construction. Sequencing was performed on Illumina MiSeq (Illumina, Paris 3, France) 2*250 bp at Get-PlaGe France Genomics sequencing platform (Toulouse, France). Sequence data cleaning, filtering, clustering into OTUs and taxa annotation was performed as described previously^64^.

### Reference transcriptomes and alignments

All the MMETSP reference transcriptomes^16^ were downloaded from Cyverse (http://www.cyverse.org). All references corresponding to unknown species were discarded. The reference corresponding to *A. minutum* was replaced by the one obtained using local strains^47^. Within each transcriptome, for each transcript, only the longest isoform was considered. When several transcriptomes per species were available (several strains or culture conditions), they were merged into a single reference, and CD-HIT-EST^65^ was used to remove homologous sequences within each reference. A total of 313 species specific reference transcriptomes, representing 213 unique genus, were considered and concatenated to build meta-references. In the first one (hereafter MetaRef1), all (313) species specific reference transcriptomes were concatenated. Reads from the meta-transcriptomic datasets were aligned to the meta-reference using the BWA-MEM aligner^66^. Samtools^67^ was used to discard reads displaying low quality alignments (MapQ<10) as well as the ones mapping to several transcripts (FLAGS < 1,000).). For each sample *i* (each meta-transcriptomic dataset), the relative abundance of the transcripts (RAT) for species *j* in sample *i* was calculated as

$$RA{T}_{ij}=\frac{{n}_{ij}}{{l}_{j}}/{\sum }_{j}\frac{{n}_{ij}}{{l}_{j}}$$

where *n*_*ij*_ corresponds to the number of reads from sample *i* mapping to species *j* reference transcriptome, *l*_*j*_ to the total length, in base, of the reference transcriptome of species *j*. The output of this first alignment is summarized in Supplementary Fig. 3a. A strong signal of this first alignment is that among the species attracting numerous environmental reads, there are several members of the same genus. This is for instance the case of the dinoflagellate genus *Alexandrium*, but also of the diatom genus *Chaetoceros* and *Thalassiosira*. Two distinct possibilities may explain this pattern. It could be observed because several species of the same genus tend to co-occur in the sampled communities. In this case, the observed pattern is ecologically relevant and should be further analyzed. Or it could be an artefact resulting from the misalignment of environmental reads belonging to a given species on the reference transcriptome of closely related species. The second meta-reference (MetaRef2) was built in order to avoid the artefactual alignments. It was composed of 219 species specific transcriptomes selected as follows. First, when a genus was represented by a single species in the MMETSP reference transcriptomes, the species was added to MetaRef2 (162 species). Second when several species belonged to the same genus, the Spearman rank correlation of the RAT obtained using MetaRef1 for each pair of species across the samples were computed. When the Spearman rank correlations were >0.75 for all pairwise comparisons within a genus, only the species with the highest maximum RAT was added to MetaRef2 (40 species). Within the genus displaying at least one Spearman rank correlation <0.75, were added to MetaRef2: 1. The species with the maximum RAT for the group of species displaying Spearman rank correlations >0.75, and 2. All the species displaying Spearman rank correlations <0.75 (25 species from 11 genus). An illustrative example is given in Supplementary Fig. 4 for the genus *Alexandrium* where a single species was selected and the genus *Chaetoceros* where three species were selected. Finally, all the reference transcriptomes displaying a total length <2.10^6^ bases were discarded (8 species). Alignment and filtering were performed as described above and the output of this alignment is summarized in Supplementary Fig. 3b. The 12 species displaying a maximum RAT > 0.03 were analyzed for species specific gene expression dynamics. Similarly, larger phylum displaying a maximum RAT > 0.03 were analyzed for phylum specific gene expression dynamics.

To estimate how the number of species in the meta-reference influenced the alignments of the reads, eight other meta-references were built using the references of the 12 selected species and 0, 25, 50, 75, 100, 125, 150, and 175 other species specific references randomly chosen from the species composing MetaRef2. Alignment and filtering were performed as described above. As presented in Supplementary Fig. 5, including numerous species in meta-references is of primary importance when dealing with environmental reads.

### Determining gene expression dynamics *in situ*

The matrix of reads aligning to MetaRef2 was used to investigate gene expression dynamics. After preliminary analyses, one sample (13/07/15) was excluded from the analyses due to poor quality. Transcripts covered by less than 10 reads on average across samples were discarded from the analyses. The dataset was normalized using Deseq. 2 Variance Stabilizing Transformation^68^. After preliminary PCA and clustering analyses, year specific batch effects were considered as negligible and were not corrected for. Transcripts with similar expression dynamics were grouped into modules of co-expressed transcripts using the WGCNA (1.51) R package^69^. A soft-thresholding power of 6, a maximum block size of 35,000 transcripts, and bicor correlation were used as parameters. Module identification was performed using dynamic tree cut with minimum cluster size of 1,000 transcripts, and modules displaying a Pearson correlation >0.75 were merged. For each expression module, WGCNA was used to extract eigengenes, which can be defined as the first principal component of the expression matrix of the detected modules. The linear relationships between the dynamic of the eigengenes and abiotic factors as well as species and phylum specific RAT was explored using a Redundancy analysis (RDA; ade4 (1.7–6) R package^70^).

### Annotation and Gene Ontology enrichment analyses

Sequence similarity of the transcripts with genes of identified function in the UniProt database was investigated using blastx with E-Value <10^−3^. The transcripts were classified in various Gene Ontology categories (GO; http://geneontology.org/) based on this result. Within each expression module, species specific GO enrichment was analyzed. More precisely, for each species displaying more than 450 transcripts in a given expression module, GO enrichment analyses were performed for GO categories involved in Biological Processes and containing >20 transcripts. Two sided Fisher exact test followed by false discovery rate adjustment were used and GO displaying an odd-ratio >3 and a q-value < 0.01 were considered as significantly enriched. The hierarchical Gene Ontology classification system leads to redundancies in the GO enrichment analysis. To take such redundancy into account, a distance between GO categories was calculated To take such redundancy into account, a distance between GO categories was calculated as

$$1-\frac{G{O}_{i}\cap G{O}_{j}}{\min (G{O}_{i},G{O}_{j})}$$

where *GO*_*i*_ is the size of the GO category *i*^46^.

## Supplementary information

Supplementary Information.

Supplementary Information 2.

Supplementary Information 3.

## Data Availability

Raw reads from the meta-transcriptomic (10.12770/9d4131da-b33b-429b-9cdd-e7325b06f7d8) and metabarcoding (10.12770/16bc16ef-588a-47e2-803e-03b4acb85dca) datasets are available.
