# Supplementary material for: Species specific gene expression dynamics during harmful algal blooms
Source: Sci Rep. 2020 Apr 10;10:6182. doi: 10.1038/s41598-020-63326-8 (PMC7148311; doi:10.1038/s41598-020-63326-8)
Supplement: Supplementary file 1 — Supplementary Information. [file 41598_2020_63326_MOESM1_ESM.pdf]

Species specific gene expression dynamics during harmful algal blooms

Gabriel Metegnier[1,2], Sauvann Paulino[1], Pierre Ramond[1,3], Raffaele Siano[1], Marc Sourisseau[1], Christophe Destombe[2], Mickael Le Gac[1]

[1]French Research Institute for Exploitation of the Sea, Ifremer DYNECO PELAGOS, 29280 Plouzané, France
[2]CNRS, Sorbonne Université, UC, UaCh, UMI 3614, Evolutionary Biology and Ecology of Algae, Station Biologique de Roscoff, CS 90074, 29688 Roscoff, France

[3]CNRS, Sorbonne Université, UMR 7144, Station Biologique de Roscoff, CS90074, 29688 Roscoff Cedex, France

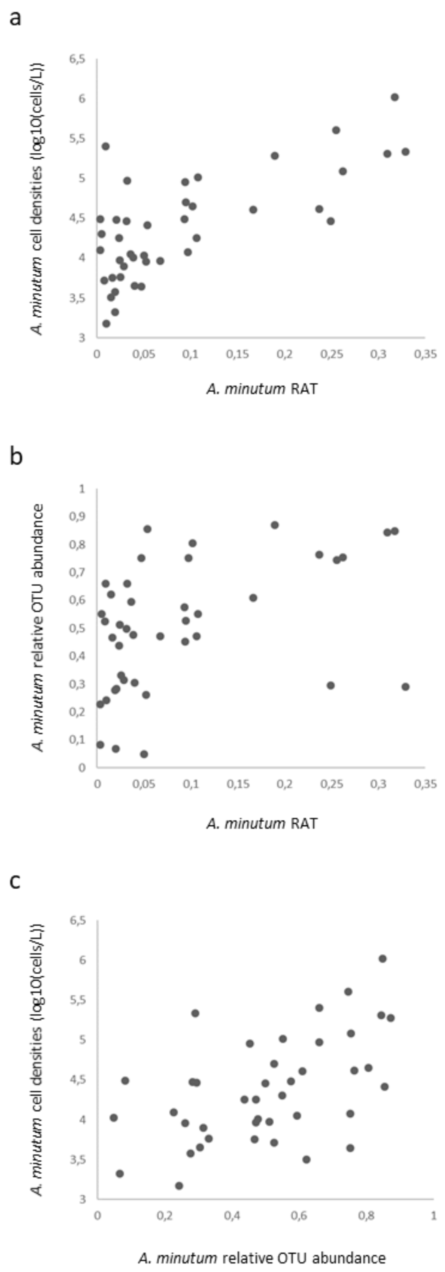

Supplementary Figure 1: a. *Alexandrium minutum* cell densities *in situ* as a function of the relative abundance of transcripts (RAT) in the meta-transcriptomic samples; b. *Alexandrium minutum* OTU relative abundances as a function of RAT; c. *Alexandrium minutum* cell densities *in situ* as a function of OTU relative abundances. Each dot corresponds to a sampling date.

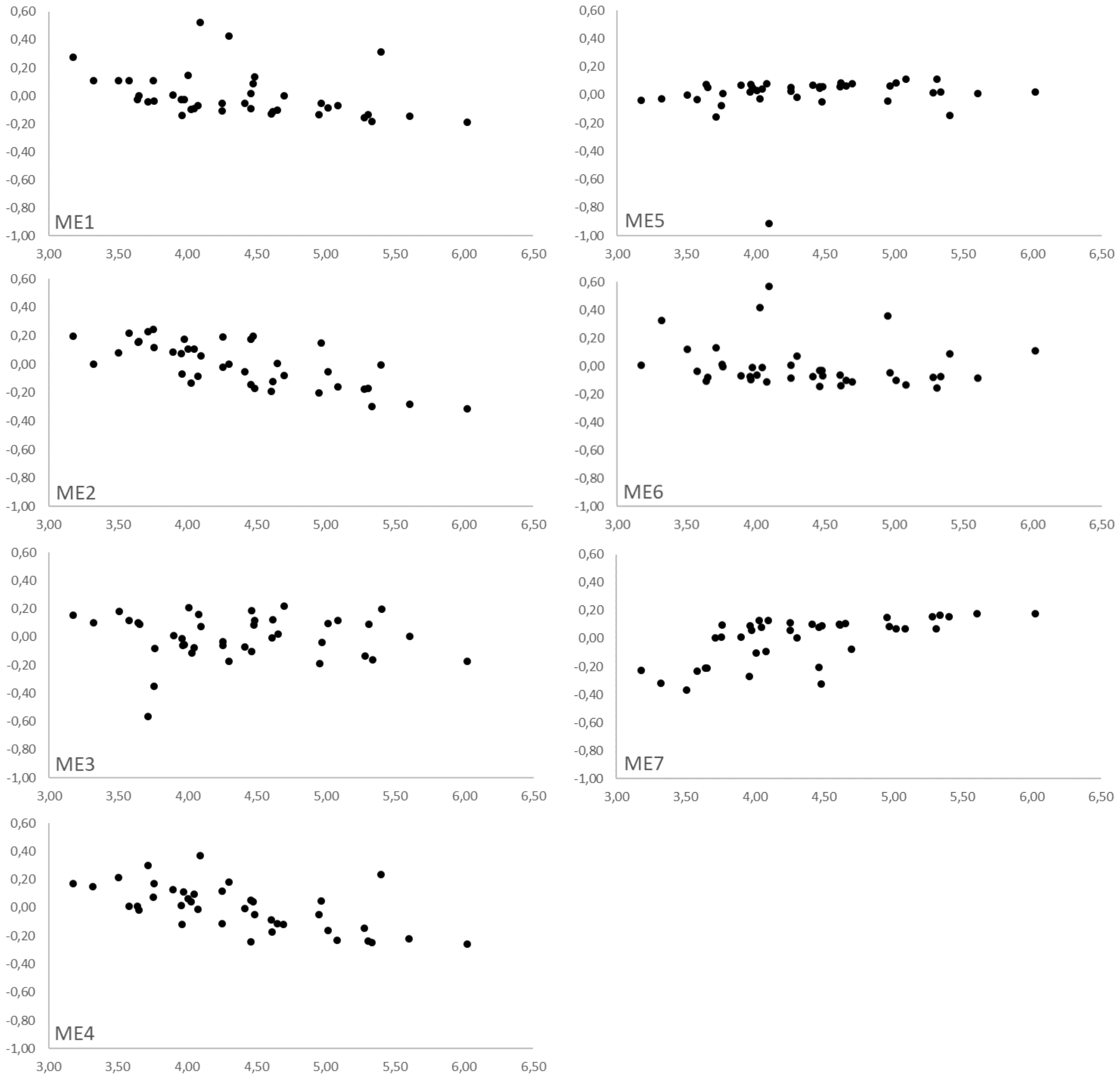

Supplementary Figure 2: Relationship between *Alexandrium minutum* cell densities (log10) *in situ* (x-axis) and the expression of the eigengenes of each expression module (y-axis) (ME1-7).

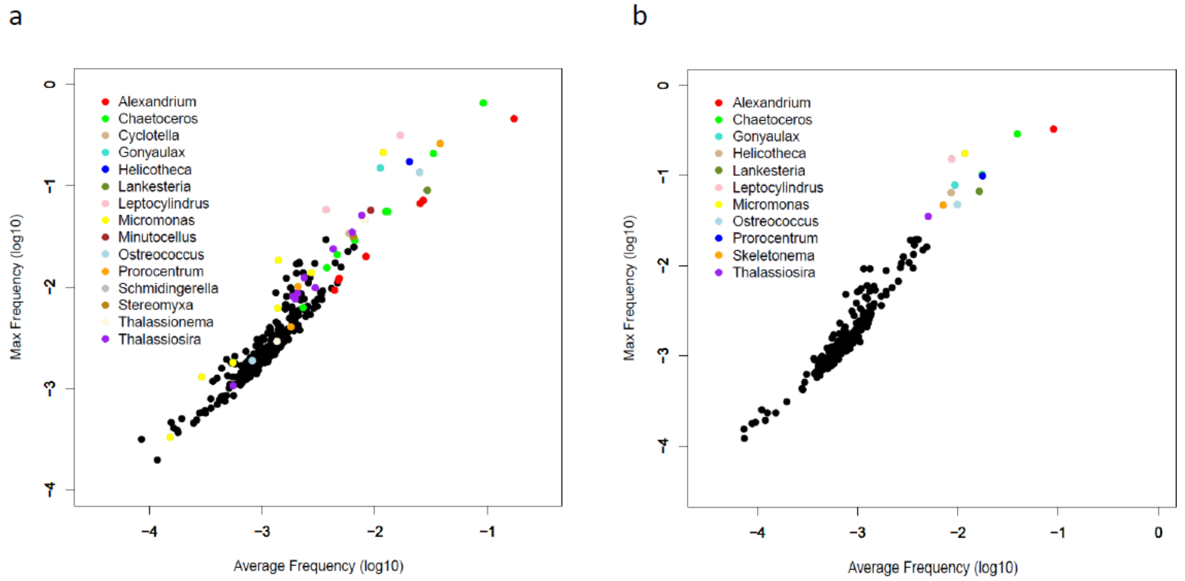

Supplementary Figure 3: Alignment of environmental reads on a. MetaRef1 and b. Metaref2. Each point corresponds to a species. Positions of the points are given based on the maximum proportion (y axis) and average proportion (x-axis), across samples, of reads aligning to the species specific reference transcriptome within the metareference. Colors indicate a. the members of genus for which at least one species displays a maximum proportion > 0.03, b. the twelve species considered for the subsequent analyses.

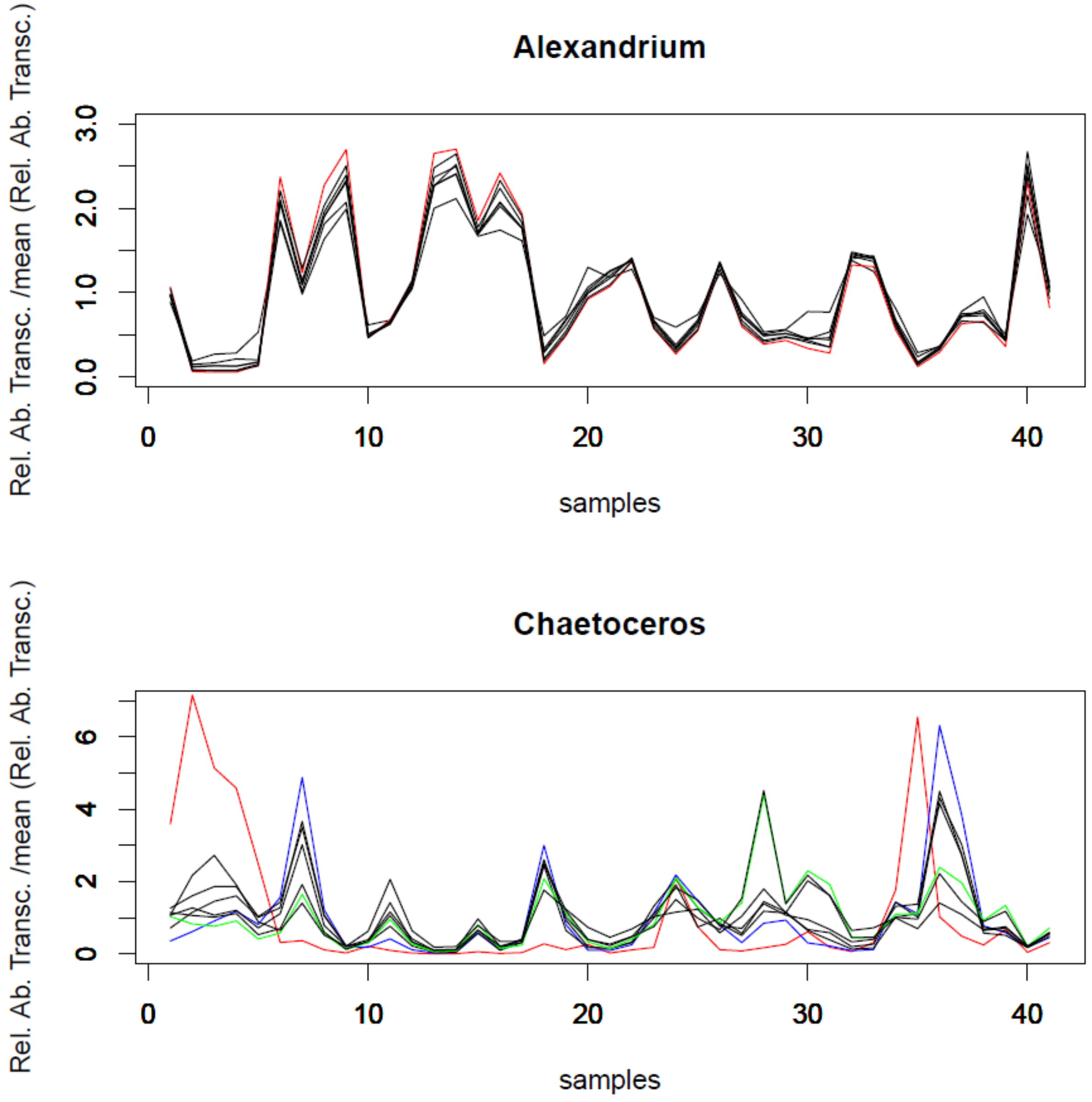

Supplementary Figure 4: For the genus *Alexandrium* and *Chaetoceros*, alignment of the environmental reads (RAT/mean RAT) on MetaRef1 across the 41 samples. Each line corresponds to a species. For *Alexandrium*, a single species (*A. minutum,* red) was selected to be included in MetaRef2. For Chaetoceros, three species (*C. curvisetus,* red; *C. cf neogracile*, blue; and *C. affinis*, green) were included in MetaRef2.

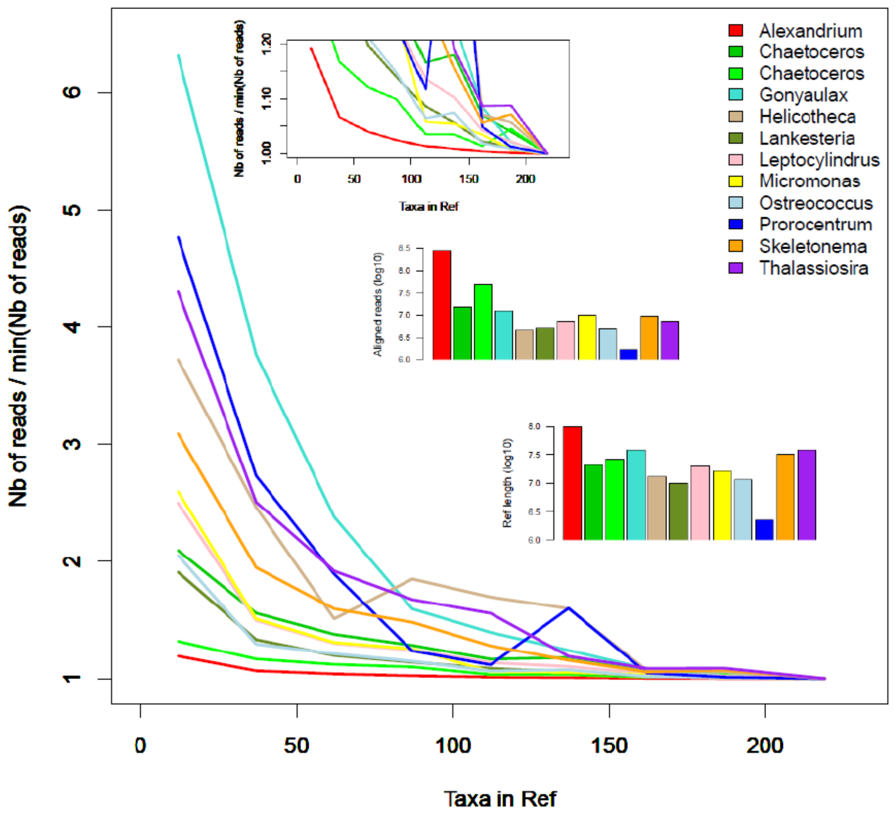

Supplementary Figure 5: Impact of the number of species specific references in the meta-reference (x axis) on the environmental read alignment on the reference of the 12 selected species (y axis; number of reads aligning to the species specific reference divided by the number of reads aligning to the species specific reference when using Metaref2 (219 species). Each color corresponds to a species. The three insets correspond from top to bottom to: 1. The magnification of the lower part of the main graph, 2. For each species, the total number of aligned reads when aligning against MetaRef2, 3. For each species, the length of the references in base. Interestingly, the magnitude of the response was extremely different across species, the two extremes being G. spinifera and A. minutum. The former species attracting six times more reads when the metareference is composed of 12 species than when it is composed of 219 species (MetaRef2) while for the latter the difference is below 0.2. We note that this huge difference can neither be explained by differences in the length of the reference transcriptomes (lower inset) nor by the number of reads aligning to a given species.
